# Development and Formative Evaluation of a Visual E-Tool to Help Decision Makers Navigate the Evidence Around Health Financing

**DOI:** 10.2196/resprot.2173

**Published:** 2012-12-21

**Authors:** Jolene Skordis-Worrall, Anni-Maria Pulkki-Brännström, Martin Utley, Gayatri Kembhavi, Nouria Bricki, Xavier Dutoit, Mikey Rosato, Christina Pagel

**Affiliations:** 1 UCL Institute for Global HealthLondonUnited Kingdom; 2 Clinical Operational Research UnitUCLLondonUnited Kingdom; 3 Save the Children UKLondonUnited Kingdom; 4 Sydesy.comLondonUnited Kingdom; 5 Centre for Anthropological ResearchUniversity of JohannesburgJohannesburgSouth Africa

**Keywords:** health care systems, financing, policy makers, software tools

## Abstract

**Background:**

There are calls for low and middle income countries to develop robust health financing policies to increase service coverage. However, existing evidence around financing options is complex and often difficult for policy makers to access.

**Objective:**

To summarize the evidence on the impact of financing health systems and develop an e-tool to help decision makers navigate the findings.

**Methods:**

After reviewing the literature, we used thematic analysis to summarize the impact of 7 common health financing mechanisms on 5 common health system goals. Information on the relevance of each study to a user’s context was provided by 11 country indicators. A Web-based e-tool was then developed to assist users in navigating the literature review. This tool was evaluated using feedback from early users, collected using an online survey and in-depth interviews with key informants.

**Results:**

The e-tool provides graphical summaries that allow a user to assess the following parameters with a single snapshot: the number of relevant studies available in the literature, the heterogeneity of evidence, where key evidence is lacking, and how closely the evidence matches their own context. Users particularly liked the visual display and found navigating the tool intuitive. However there was concern that a lack of evidence on positive impact might be construed as evidence against a financing option and that the tool might over-simplify the available financing options.

**Conclusions:**

Complex evidence can be made more easily accessible and potentially more understandable using basic Web-based technology and innovative graphical representations that match findings to the users’ goals and context.

## Introduction

Against a background of calls for robust domestic health financing to attain and sustain increased service coverage [1], financing choices have seldom been so complex. The gradual removal of user fees is leaving a policy and funding vacuum in many low and middle income countries [2-4].

It is probable that the ideal health financing approach is a nuanced mix of methods appropriate to a given economic, social, political, and epidemiological context, and designed to best meet the most urgent health system priorities without significant negative consequences [5-7]. However, the pragmatic reality is that the process of defining and implementing this balance may create confusion and policy paralysis. This paralysis may be further exacerbated by the breadth and heterogeneity of a relatively fast-growing body of evidence.

The last comprehensive review of the evidence on domestic health financing was conducted more than 7 years ago by Palmer et al [5], with Lagarde and Palmer [8] more recently reviewing the evidence on user fees. We build on this valuable evidence base in 2 ways- firstly we collate the evidence on a wide range of health financing methods to reflect the most current learning, and secondly we report on a broader set of impacts including service quality, poverty and equity, and revenue generation. Such reviews are generally not tailored to the needs of policy makers and other common users, and there is a recognized need to make that evidence more accessible [9].

In this paper we describe our development of an e-tool that summarizes the available literature quickly and easily in both graphical and tabular form, with the added ability to access the underlying evidence from anywhere within the tool. We describe the methods used to extract the literature, synthesize it into the e-tool and evaluate the overall usefulness of the final product. In the results we describe the final e-tool and briefly summarize the key findings of the literature review to provide the reader with a sense of the depth and complexity of the evidence incorporated into the Web-based platform. Finally, we reflect on the feedback from early users of the tool to highlight critical benefits, limitations, and lessons learned.

## Methods

This section outlines the methods used for the literature review and synthesis of that evidence, the methods used to construct the e-tool using the review material, and the methods used to generate and synthesize user feedback.

### Literature Review and Synthesis

Literature review methods were adapted from the EPPI-Centre [10] and Greenhalgh et al [11]. The following databases were searched using a consistent and comprehensive set of search terms: PubMed, Web of Science (Science Citation Index and Social Science Citation Index), Journal Storage (JSTOR), and Science Direct. References of articles retrieved from the initial search were then hand-searched. Websites of international organizations including the World Health Organization, World Bank, International Labour Organization, the United Nations, the United Nations Children's Fund, and the Social Science Research Network were also searched for relevant publications. The search was limited to papers published between January 1995 and June 2010.

In the context of our review, a heath financing mechanism (also referred to as method or tool) is defined as a mechanism intended to raise domestic revenue for health including national/government/social health insurance, taxation, community-based insurance, private insurance, user fees, and equity funds. Papers were only included if published in peer-reviewed journals, available in English, focused on low and middle income countries, and specifically evaluated or discussed the outcomes of at least one health financing method. Opinion papers, editorials, conference proceedings, and letters to the editor were excluded. Articles discussing the potential for the implementation of a method, evaluating willingness to pay or providing overviews or descriptions of health financing programs without discussing or evaluating the outcomes of implemented programs, were similarly excluded. Papers were not excluded on the basis of study design as research in this field uses a wide range of qualitative and quantitative methods. Similarly, papers were not scored on any quality of research metric as there are few widely accepted criteria for doing so in the field of economics that would span both qualitative and quantitative research outputs. Our intention was to avoid value judgments and conduct as inclusive a review as possible, given that users of the e-tool are able to identify the source of any evidence simply by hovering over a dot, and can access the paper itself with a single click.

From an initial shortlist of 151 papers, a total of 78 articles were included in the final database. A thematic analysis of the papers included in the final review was used to construct a data extraction form to systematically extract relevant information from each article reviewed. A subset of extracted data was independently reviewed by 3 researchers (JSW, GK, APB) to confirm that the extracted data accurately reflected the papers’ content. This review thus takes a rigorous and systematic approach of searching, data extraction and synthesis, resulting in comprehensive findings that provide a valuable contribution to the existing body of knowledge in this area.

The possible outcomes of a health financing method were condensed into 5 domains or goals as shown in Table 1. For each article, we summarized the outcome of the financing mechanism on each of the goals in Table 1 by assigning 1 of 5 qualitative scores: "evidence against", "some evidence against", "no evidence of impact", "some evidence for", and "evidence for". We explicitly recorded if a goal was not considered as part of the study, to reflect where evidence was lacking.

Authors (ACP, JSW and APB) independently used the extracted summary information to assign impact or outcomes scores based on the conclusions of included studies. For instance, if a study concluded that a health financing tool reduced out-of-pocket payments then this was considered “evidence for” poverty reduction. If the authors had reported only slight reduction in these payments this would have been considered “*some* evidence for” poverty reduction. The 3 authors assigning scores then compared their findings, and resolved any discrepancies through group discussion. The final scores were transferred to Microsoft Excel to form the basis of the e-tool. During this process we did not make any judgements about the validity of conclusions regarding impact or program effect, nor did we make any assessment of study quality. Our scores are intended purely as a visual summary and an aid to further investigation. The decision to have a qualitative rather than a quantitative impact score is intended to make this clear to the user. The evidence available was qualitative, descriptive, and heterogeneous.

**Table 1:** Possible goals of a health financing policy.

Goal	Brief description
Promote equity	Incorporates references to relative poverty reduction, equity, the distribution of disease burden (eg, disability-adjusted life-years), the distribution of the financial burden (eg, the incidence of catastrophic health spending), and risk pooling.
Reduce poverty	Refers to changes in absolute poverty.
Improve quality	Refers to service quality and changes in health outcomes that may be a consequence, or indicator, of improved quality.
Generate revenue	Refers to either absolute or relative revenue generation at any tier of health service delivery. Assumes that increased revenue generation or retention is the desired outcome.
Increase use	Refers to the quantity of health services demanded, access to care and utilisation of health services.

### Converting the Findings into a Scattar Plot

As the evidence for any financing method varied significantly by context, we sought to inform the user how closely the countries analyzed in each study matched their own. To this end, a subset of the study team (JSW and CP) produced an initial long list of country indicators relevant to health financing policy, and on which countries could be matched. It was considered important that indicators be easily available for most countries and transparent to users so that understanding these data did not constitute a barrier to using the tool. The long list was then reduced to a final set of eleven indicators judged by the full study team to provide independent information relevant to financing policy and appropriate for matching contexts.

**Figure 1:**
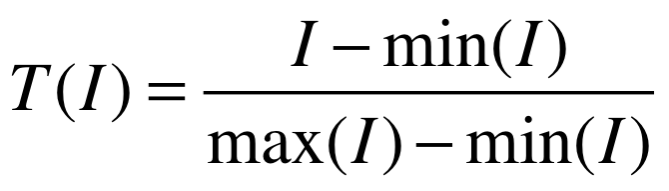
Equation to define T(I).

**Figure 2:**
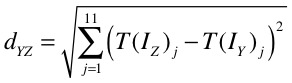
Equation to define the distance function.

These indicators, given in Table 2, were used to match a user’s country to the evidence. We transformed the value of each indicator, *I*, into a value, *T(I)*, between 0 and 1 according to Figure 1, where the maxima and minima were taken across all low and middle income countries. Each country is thus characterized by a set of 11 values between 0 and 1, {*T(I)*_*j*_}, where j runs from 1 to 11. These values can be thought of as a single point on an 11-dimensional graph, and we use the 11-dimensional distance, *d*_*YZ*_, given in Figure 2, between 2 points as an estimate of how closely 2 countries Y and Z are matched.

Multimedia Appendix 1 gives a graphical example of the distance between 2 countries for 3-dimensions, and a plot of how 7 countries are placed in relation to an example country. The *context matching* criteria were checked for a subset of countries to ensure there was face validity but there was no formal validation of the measure. Again, the use of this measure was to aid the user to visually sort the available evidence (comparing *more closely matched* to *less closely matched* evidence). The exact ranking of countries with respect to their “context match” of another country was not intended to be an important output.

**Table 2:** Country indicators used for context matching.

Indicator	Source (2008 data)
Health expenditure per capita ($)	World Bank
Maternal mortality ratio	World Bank
Under 5 mortality rate	World Bank
HIV prevalence	World Bank
Malaria incidence	United Nations
Education index	United Nations Development Program
GDP ($)	World Bank
Life expectancy at birth	United Nations Development Program
Proportion of the population living in an urban environment	World Bank
Proportion of the population living on less than $1.25 a day at 2005 international prices	World Bank
Population (log, base 10)	World Bank

**Figure 3:**
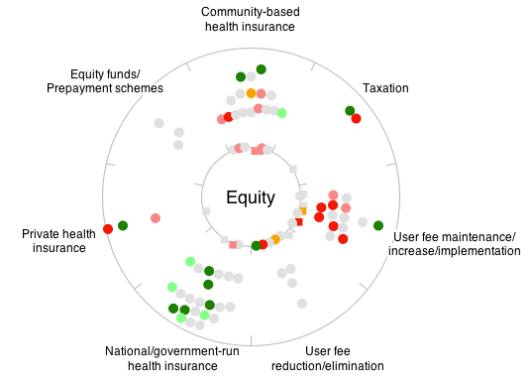
Example of a 'scattar' plot showing the available evidence of impact of each financing tool on promoting equity in Uganda. Each dot represents a single study and its colour represents the reported impact on the specified goal.

The next stage in the process was to explore ways of displaying the available evidence in an intuitive and informative way. After several iterative discussions within the development team and experimentation with different ways of displaying the data, we developed a *scattar* plot, incorporating elements of both scatter and radar charts. Each such display focuses on one goal of a health financing policy and shows the evidence available for all 7 health financing mechanisms in the context of the (user-specified) country of interest. Figure 3 shows an example of how this would apply to the promotion of equity in Uganda.

Literature reviews are differentiated from evaluation-style studies in an attempt to reduce the risk of double-counting evidence. Review studies are thus shown in square dots on the inner ring while all other studies are represented as round dots. The distance of the dots from the inner ring within the *scattar* plot indicates how closely the countries within each study match the user-specified country (eg, Uganda in Figure 3). Round dots on the inner circle correspond to studies that consider exactly the country chosen by the user.

The colour of each dot indicates the impact of the financing mechanism on the chosen goal (eg, promoting equity) as reported in the cited study. These colours range from green for positive impact to red for negative impact, with orange indicating that there was definite evidence of no impact. Dots coloured in grey correspond to studies that considered a given health financing tool but did not consider the impact on the goal under consideration. We felt it was important to include these on the graphical display as they give an indication of where evidence is lacking. All possible plots would always have the same number of dots (since all studies are shown on every plot). The position of the dots within the ring will change according to the user-specified country and the colour of the dots will change according to the user-specified goal.

### Eliciting and Analysing Feedback from Users

In September 2011, the tool was launched by Save the Children, through an email to policy makers, international agencies, and researchers. Feedback was collected using a mixed methods approach including an online survey (n=19) and semi-structured key informant interviews (n=8). Due to the small quantitative sample, the results will be treated qualitatively and only percentages over 50% will be reported in the findings. The small sample of survey respondents is acknowledged as a limitation of this study and it is further acknowledged that the survey respondents may not constitute a random or representative sample of potential users of the tool. However, despite the small number of respondents to the survey, we were able to obtain responses from a wide spectrum of stakeholders including government policy advisors, consultants, staff from national and international non-governmental organisations (NGOs), as well as students and academics. Key informants included respondents from:

- United Nations Children’s Fund
- World Health Organisation
- The UK Department for International Development
- The Bill and Melinda Gates Foundation
- Imperial College London
- NGOs including; Research 4 Development, Oxfam, World Vision, Save the Children UK (Zimbabwe, South Africa and Ethiopia country teams)

The findings from the key informant interviews were synthesised using thematic analysis.

## Results

### Summary of Literature Review Results

From an initial shortlist of 151 papers, a total of 78 articles were included in the final analysis. The health financing methods included in the tool, together with the number of papers providing evidence of impact of this tool on a health financing goal are as follows:

- Equity funds and discount cards (2 papers)
- Tax-funded systems (2 papers)
- Private health insurance (4 papers)
- User fees - which we segmented into: (1) the implementation, increase, or maintenance of fees (21 papers), and (2) the reduction or elimination of fees (10 papers)
- Community-based health insurance (CBHI) (24 papers)
- National/government-run health insurance (NHI) including social health insurance (25 papers)

This review provided an opportunity to update the evidence on a wide range of health financing methods, generating a number of key insights. Firstly, the breadth of the review highlighted that the weight of evidence is unevenly distributed between health financing methods. In particular, little has been written since 1995 about the impact of tax-based financing despite the recognition that most countries that have achieved universal risk protection have done so through tax financed systems. Secondly, no single method emerges as having the greatest positive impact on utilisation, service quality, equity and risk pooling, poverty, and revenue, although the tool highlights the positive and negative impacts of each method on these goals. Importantly, no financing method effectively removes or redresses the indirect costs faced by the poor when accessing health services. Thirdly, the small body of evidence on private health insurance raises concerns about adverse, unintended consequences. Fourthly, the large body of evidence on CBHI and NHI offers a mixed perspective on their use. Compared to Palmer et al [5] the new evidence on CBHI reviewed in our paper indicates, in general, that there is an increase in access and utilisation of health services amongst member households, although this increase may not be among the poorest. The new evidence on NHI indicates that in many cases, coverage of these schemes does not reach the most vulnerable groups of a population and utilisation of services remains low outside major urban centres due a lack of health facilities.

Finally, compared to Lagarde and Palmer [8], a substantial number of new papers on the implementation of user fees have been included in this review without adding very significant new insights about any positive impact. User fees did contribute to revenue generation, but this varied significantly between settings. Overall the implementation of user fees had a negative impact on equity. The evidence we reviewed about the impact of the removal of user fees on quality of services was mixed, and confounded by simultaneous health system strengthening measures. The narrow evidence base on the removal of user fees shows potential for improvements in equity and use.

**Figure 4:**
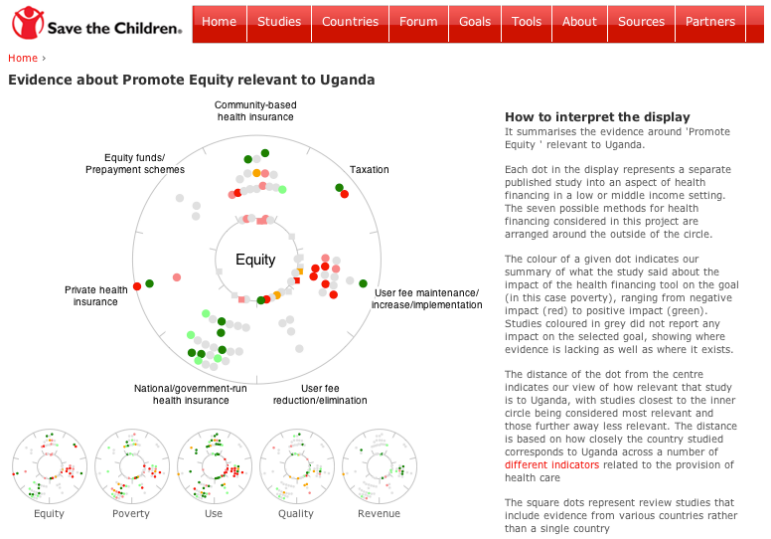
Screenshot of the website’s graphical summary. As for Figure 1, the chosen country is Uganda and the chosen goal is "Promote Equity".

In Figure 4, the user can quickly see that there is most evidence around national and community health insurance and the implementation of user fees. There is some evidence directly related to Uganda (round dots on the inner circle). The large number of gray dots shows that many studies did not consider the impact of the health financing method on equity. Looking at the colours of the dots, the evidence suggests that national health insurance schemes have a positive impact on equity, user fee implementation a negative impact, and that the evidence on community-based health insurance is mixed. The 2 studies where community-based health insurance had a positive impact on equity are least well matched to Uganda's context (the dots are close to the outer ring), but 2 community-based health insurance studies directly related to Uganda show some evidence of a negative impact.

### The Tool and the Scattar Plots

After consulting informally with potential users, it was decided that the most accessible platform for the tool would be a website where users could navigate easily between different countries and goals. Additional advantages of an online tool were that it presented a familiar interface to users-it would be easy to access, it would be easy to update and maintain version control, and it provided an ideal environment to cross-reference between studies, countries, tools and goals. The website was registered under Save the Children’s branding, since they funded and commissioned this work [12].

On the site, the user begins by choosing the country and health financing goal, before being taken to a page showing the relevant *scattar* plot (see Multimedia Appendix 2 for a screencast). We thought it important that the user is asked to choose a goal first to highlight the fact that while more than one goal may be of interest, the impact of a health financing mechanism is not necessarily the same for all 5 goals. On the right hand side of the page showing the *scattar* plot is an explanation of how to interpret the display (Figure 4). Thumbnails of all 5 goals are given at the bottom of the main plot allowing the user to switch easily between goals and giving an immediate visual impression of the distribution of the evidence.

**Figure 5:**
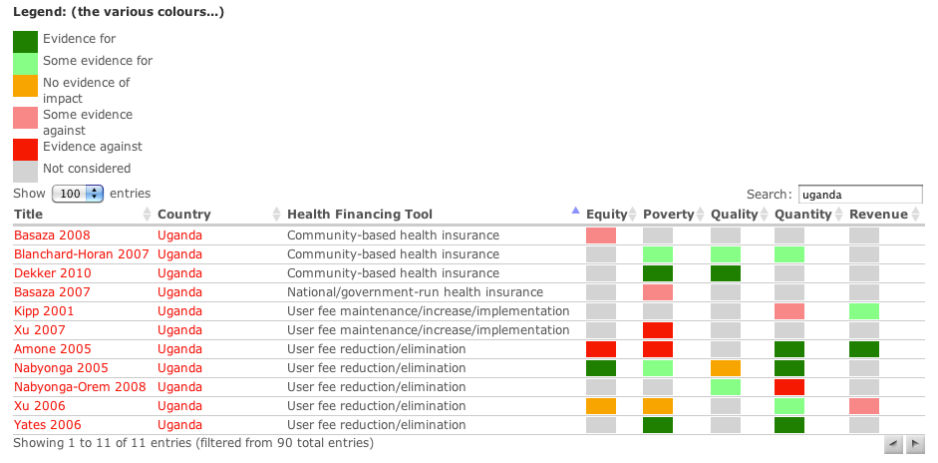
Screenshot of the list of studies showing summary of impact. Any search term could be used (here: Uganda), or the gray arrows at the top of each column used to sort the entire list. The text in red represents links to the extended information on the given studies and country.

The user always has access to the evidence on which the summaries are based. Hovering over any dot displays the authors, paper title, and country studied (Multimedia Appendix 2). Selecting a dot will bring the user to a separate page in a new tab with the full reference, a link to the publisher, the published abstract, and our summary of the evidence of impact for all 5 goals. There are also separate webpages within the site providing a searchable and sortable list of studies used, with summaries of impact (Figure 5) and context-matching indicator values for each country.

In several places on the website, the simple and qualitative nature of the impact assessment of studies is stressed, as well as the overall aim to help users navigate the evidence. We stress in the online explanations that our summaries are not considered a suitable basis for action without further investigation. By making it easy for the user to access the evidence used for this tool from many places on the site and according to different criteria (eg, country, goal, health financing mechanism), we hope that we have made clear its exploratory intent. In a sense the website is intended as a directional magnifying glass to help users identify the evidence that they might find useful quickly according to their particular questions and interest.

### Feedback from Users

Feedback from the survey was very positive, with 90% (17/19) of respondents stating that the website was either very or extremely easy to navigate. The most commonly used features of the website were the graphical summaries (53%, 10/19) and the country information (68%, 13/19). Respondents particularly liked the graphical summaries and the summary tables and found the colour-coded system useful and intuitive. Although most respondents had not visited the website with a particular question in mind, 90% (17/19) of respondents did find the website helpful and 63% (12/19) of respondents are either very or extremely likely to recommend the website to others.

Findings from the in-depth interviews supported this positive view, although a number of constructive criticisms and suggestions were proposed. Firstly, there was concern that a lack of evidence might be construed as evidence against a financing option. There were calls for the tool to more clearly distinguish between negative evidence and lack of evidence. Secondly, respondents expressed a concern that the tool might encourage users to take an over-simple view of financing options as it does not offer the option of integration or mixing financing methods and does not take into account of the context in which they are implemented. This is indeed a limitation of the tool imposed on the production team by the paucity of evidence on mixed methods financing approaches. Thirdly, it was suggested that the power of the tool is not immediately obvious and that a guide or manual available online might be a useful resource. Finally, one respondent suggested that new literature might already have emerged which would warrant inclusion on the site. This latter point was considered critical by the team producing the tool and has been a consideration from the outset of the project.

## Discussion

The health financing debate is moving on in low and middle income countries, from asking whether user fees should be removed, to exploring how to finance healthcare to achieve universal risk protection whilst achieving equity, efficiency, and quality of care. A conventional literature review will always be challenged by the breadth and complexity of the evidence on health financing, and the need to condense that evidence into a single journal article. Additionally, a published literature review is not necessarily the easiest way for a policy maker, or other user outside of academia, to access the evidence. We have developed a new e-tool that helps users (whether policy makers, NGO workers, or academics) to navigate the complex evidence by focusing on a single intelligent snapshot of the literature. Our tool is easy to access, and provides a rapid search of the evidence by country and goal. The tabular summary also allows the user to search the evidence according to a variety of criteria such as finance mechanism, country, or impact among others. Given its structure and Web implementation, the tool is also designed to be easily updated as new evidence emerges and country indicators change. These positive aspects of the tool were mentioned in the feedback received from early users during the evaluation stage of this process.

That said, to structure the tool in a comprehensible and navigable form, it was necessary to make certain simplifications or groupings within the evidence–particularly with regards to the assignment of impact. We attempted to minimize the loss of detail by allowing users to link through to the original articles. This loss of complexity is a common tension when synthesising evidence and is highlighted in the feedback from users as a risk of our tool (ie, that users might think only in the discrete and mutually exclusive categories of health financing options presented in the tool, forgetting that a mix of financing methods might be the most appropriate solution for their context). Understanding more fully how users interpret and use the output of a tool such as this may be a rich area for future research. However, while we needed to simplify the evidence somewhat, we would argue that this form of e-tool requires less simplification than a static journal article. We would also argue that our tool is evidence based, and the failure of our tool to shed light on the benefits of mixing financing methods reflects a failure of the academic literature to shed light on this option.

At every stage in building the tool we tried to be transparent about our assumptions and methods and always provide links for the user to the original evidence. Thus, the tool is intended to be used for exploration, allowing users to drill quickly down to the evidence most relevant to their needs, and not to make any finite recommendations for policy or health financing mechanism for a given country. While the tool has only been recently launched, it is hoped that it will become an important supplement to the existing literature on health systems financing. We also hope that this methodology could be used to bridge the gap between academic knowledge and practice for other complex policy questions.

## Multimedia Appendix 1

Context matching of the studies.

## Multimedia Appendix 2

Screencast of how a user might navigate the tool.

## Abbreviations

CBHI: community-based health insurance
NGO: non-governmental organisation
NHI: national or government-run health insurance

## References

[1] Evans DB, Etienne C (2010). Health systems financing and the path to universal coverage. Bull World Health Organ.

[2] Borghi J, Ensor T, Somanathan A, Lissner C, Mills A, Lancet Maternal Survival Series steering group (2006). Mobilising financial resources for maternal health. Lancet.

[3] Gilson L, McIntyre D (2005). Removing user fees for primary care in Africa: the need for careful action. BMJ.

[4] James CD, Hanson K, McPake B, Balabanova D, Gwatkin D, Hopwood I, Kirunga C, Knippenberg R, Meessen B, Morris SS, Preker A, Souteyrand Y, Tibouti A, Villeneuve P, Xu K (2006). To retain or remove user fees?: reflections on the current debate in low- and middle-income countries. Appl Health Econ Health Policy.

[5] Palmer N, Mueller DH, Gilson L, Mills A, Haines A (2004). Health financing to promote access in low income settings-how much do we know?. Lancet.

[6] Kutzin J, Ibraimova A, Jakab M, O'Dougherty S (2009). Bismarck meets Beveridge on the Silk Road: coordinating funding sources to create a universal health financing system in Kyrgyzstan. Bull World Health Organ.

[7] Normand C, Thomas S, Quah S (2008). Health care financing and the health system. International Encyclopedia of Public Health.

[8] Lagarde M, Palmer N (2008). The impact of user fees on health service utilization in low- and middle-income countries: how strong is the evidence?. Bull World Health Organ.

[9] Rosenbaum SE, Glenton C, Wiysonge CS, Abalos E, Mignini L, Young T, Althabe F, Ciapponi A, Marti SG, Meng Q, Wang J, la Hoz Bradford AM, Kiwanuka SN, Rutebemberwa E, Pariyo GW, Flottorp S, Oxman AD (2011). Evidence summaries tailored to health policy-makers in low- and middle-income countries. Bull World Health Organ.

[10] EPPI-Centre (2007). EPPI-Centre methods for conducting systematic reviews.

[11] Greenhalgh T, Robert G, Macfarlane F, Bate P, Kyriakidou O, Peacock R (2005). Storylines of research in diffusion of innovation: a meta-narrative approach to systematic review. Soc Sci Med.

[12] (2011). Save the Children Federation, Inc.

